# High-precision MRI of liver and hepatic lesions on gadoxetic acid-enhanced hepatobiliary phase using a deep learning technique

**DOI:** 10.1007/s11604-024-01693-2

**Published:** 2024-11-11

**Authors:** Haruka Kiyoyama, Masahiro Tanabe, Keiko Hideura, Yosuke Kawano, Keisuke Miyoshi, Naohiko Kamamura, Mayumi Higashi, Katsuyoshi Ito

**Affiliations:** Department of Radiology, Yamaguchi University Graduate School of Medicine, 1-1-1 Minami-Kogushi, Ube, Yamaguchi 755-8505 Japan

**Keywords:** Compressed sensing, Gadoxetic-acid, Hepatobiliary phase, Fast 3D mode wheel and PIQE

## Abstract

**Purpose:**

The purpose of this study was to investigate whether the high-precision magnetic resonance (MR) sequence using modified Fast 3D mode wheel and Precise IQ Engine (PIQE), that was collected in a wheel shape with sequential data filling in the k-space in the phase encode-slice encode plane, is feasible for breath-hold (BH) three-dimensional (3D) T1-weighted imaging of the hepatobiliary phase (HBP) of gadoxetic acid-enhanced MRI in comparison to the compressed sensing (CS) sequence using Advanced Intelligent Clear-IQ Engine (AiCE).

**Methods:**

This retrospective study included 54 patients with focal hepatic lesions who underwent dynamic contrast-enhanced MRI. Both standard HBP images using CS with AiCE and high-precision HBP images using modified Fast 3D mode wheel and PIQE were obtained. Image quality, signal-to-noise ratio (SNR), and contrast-to-noise ratio (CNR) were evaluated using the Wilcoxon signed-rank test. *p* values of < 0.05 were considered to be statistically significant.

**Results:**

Scores for image noise, conspicuity of liver contours and intrahepatic structures, and overall image quality in high-precision HBP imaging using modified Fast 3D mode wheel and PIQE were significantly higher than those in HBP imaging using CS and AiCE (all *p* < 0.001). There was no significant difference in the presence of artifact and motion-related blurring. There were no significant differences between the sequences in SNR (*p* = 0.341) or CNR (*p* = 0.077). The detection rate of focal hepatic lesions was 71.4–85.3% in CS with AiCE, and 82.2–95.8% in modified Fast 3D mode wheel and PIQE.

**Conclusion:**

A high-precision MR sequence using a modified Fast 3D mode wheel and PIQE is applicable for the HBP of BH 3D T1-weighted imaging.

## Introduction

Breath-hold (BH) fat-suppressed (FS), three-dimensional (3D), and T1-weighted gradient echo (GRE) sequences have been utilized for the detection of focal hepatic lesions, including metastatic lesions and hepatocellular carcinoma, in the hepatobiliary phase (HBP) of magnetic resonance (MR) imaging using gadoxetic acid (Gd-EOB-DTPA) [1–4]. High spatial and contrast resolution imaging is expected for the detection of small hepatic lesions, but its image quality has not yet been fully achieved. With the recent development of MR technology, several techniques have been applied to liver imaging to reduce imaging time, including the compressed sensing (CS) technique using nonlinear iterative reconstruction from variable-density random undersampling of k-space data [5, 6]. The reduced acquisition time with CS can be used in combination with deep learning denoising techniques (Advanced Intelligent Clear-IQ Engine [AiCE]) to improve spatial resolution while maintaining the signal-to-noise ratio (SNR) [7]. The recently developed Fast 3D mode wheel sequence is an alternative technique that can reduce the imaging time using signal collection in a wheel shape from the center of a fan-shaped segmented k-space in the phase encode-slice encoding plane [8]. More recently, a modified Fast 3D mode wheel sequence was available in which the signal was collected in a wheel shape with sequential data filling in the k-space in the phase encode-slice encode plane. Additionally, the Precise IQ Engine (PIQE) technique has been developed [9], in which low-SNR, low-resolution images are reconstructed to achieve a high-SNR, high-resolution imaging using deep learning denoising and up-sampling. Thus, high-precision MR imaging may be achieved by a combination of the modified Fast 3D mode wheel and PIQE without increasing the acquisition time. PIQE can technically be used in combination with the modified Fast 3D mode wheel but not with CS. Therefore, the purpose of this study was to investigate whether the high-precision MR sequences using the modified Fast 3D mode wheel and PIQE is feasible for BH 3D T1-weighted GRE imaging of the HBP in patients with focal hepatic lesions in comparison to the CS sequence using AiCE.

## Materials and methods

### Study population

**Fig. 1:**
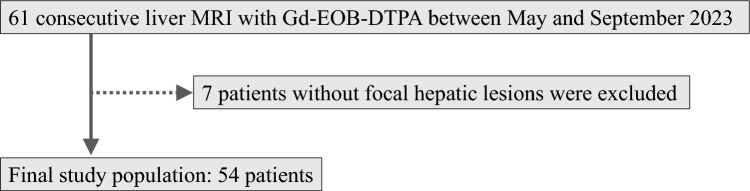
Flowchart of patient selection

Our institutional review board approved this retrospective study, and the institutional ethics committee waived the requirement for informed consent. A total of 61 consecutive patients who underwent liver MRI with Gd-EOB-DTPA between May and September in 2023 were identified. Among these, 54 patients with focal hepatic lesions were included in this study. The patient population consisted of 36 men and 18 women (mean age, 70.5 years; range, 18–93 years) (Fig. 1). The reasons for liver MRI were surveillance or follow-up for hepatocellular carcinoma in cirrhosis (n = 24), focal hepatic lesions detected on CT (n = 9), and evaluation of metastasis (n = 21). Demographics of study population are shown in Table 1.

**Table 1:** Demographics of study population

Characteristics	Patients, n (%)
Total number of patients	54
Man/woman	36 (67) /18 (33)
Age (years)^a^	70.5 ± 13.0
Underlying liver disease	
Chronic hepatitis B	9 (17)
Chronic hepatitis C	9 (17)
Non-B, non-C liver cirrhosis	4 (7)
Alchoholic liver cirrhosis	3 (6)
Autoimmune hepatitis	1 (2)
Healthy liver	28 (52)
Child–Pugh score	
5	37 (69)
6	12 (22)
7	3 (6)
8	1 (2)
12	1 (2)

^a^ Data are means ± standard deviations

### MRI technique

MRI was performed in all patients using a 3-T MR system (Vantage Centurian; Canon Medical Systems, Otawara, Japan). BH dynamic contrast-enhanced (CE) 3D T1-weighted GRE imaging with FS was conducted before and after the intravenous administration of Gd-EOB-DTPA (0.1 mg/kg, EOB Primovist; Bayer Phama, Osaka, Japan). The contrast material was injected using a power injector at rate of 1.0 mL/s, and flushed with 20 mL of saline. Multiphasic images were obtained during the arterial, portal venous, transitional, and hepatobiliary phases after intravenous injection. HBP images with FS using CS and AiCE were acquired 20 min after bolus injection, and then high-precision HBP imaging with FS using the modified Fast 3D mode wheel and PIQE was performed immediately after HBP imaging using CS and AiCE. The MRI parameters for HBP imaging using CS and AiCE and high-precision HBP imaging using the modified Fast 3D mode wheel and PIQE are shown in detail in Table 2. In addition, BH multi-shot T2-weighted fast spin-echo (FSE) images with FS (repetition time [TR]/echo time [TE]: 4000/90; flip angle: 90°; slice thickness: 5 mm; field of view [FOV]: 300 × 360), BH single-shot T2-weighted FSE images with FS (TR/TE, 17,000/60; flip angle, 90°; slice thickness, 5 mm; FOV, 300 × 360), and respiratory-triggered diffusion-weighted images (DWI) (TR/TE: 2300/42; slice thickness: 6 mm; FOV: 280 × 380) were obtained between the transitional phase and the hepatobiliary phase as part of the routine liver MRI protocol.

**Table 2:** MR parameters for standard HBP images using CS and AiCE and high-precision HBP imaging using modified Fast 3D mode wheel and PIQE

MR Parameters	CS and AiCE	modified Fast 3D and PIQE
TR/TE (msec)	3.4/1.3	3.4/1.3
Flip Angle (degrees)	12	12
Bandwidth (Hz/pixel)	781	781
Slice thickness/gap (mm)	3/0	3/0
FOV (mm^2^)	320 × 380	320 × 380
Voxel size (mm)	0.59 × 0.54 × 3	0.42 × 0.42 × 3
Acceleration factor	2.5/1.4	2/1.1
Number of excitations	1	1
Fat suppression	CHESS	CHESS
AiCE	AiCE 5, adjust 1.8	AiCE 4, adjust 1.8
Scan time (sec)	21	20

*HBP* hepatobililary phase, *CS* compressed sensing, *AiCE* Advanced Intelligent Clear-IQ Engine, *PIQE* Precise IQ Engine, TR repetition time, *TE* echo time, *FOV* field of view

### Qualitative analysis

Three radiologists with 8, 21, and 35 years of experience in abdominal MRI, respectively, reviewed images from both the standard HBP using CS and AiCE and the high-precision HBP using the modified Fast 3D mode wheel and PIQE. The radiologists were blinded to the MR parameters, laboratory data, patient histories, and final diagnoses. Three reviewers independently scored the following parameters using a 5-point rating scale: image noise (1 = nondiagnostic due to severe noise, 2 = moderate noise causing substantial impact on the diagnosis, 3 = mild noise causing little impact on the diagnosis, 4 = minimal noise, 5 = no noise), the presence of artifacts and motion-related blurring (1 = nondiagnostic due to severe artifacts, 2 = moderate artifacts causing substantial impact on the diagnosis, 3 = mild artifacts causing little impact on the diagnosis, 4 = minimal artifacts, 5 = no artifacts), conspicuity of liver contours and intrahepatic structures (1 = mostly invisible, 2 = partly invisible, 3 = unclearly visible, 4 = almost clearly visible, 5 = clearly visible), and overall image quality (1 = non-diagnostic, 2 = poor, 3 = acceptable, 4 = good, 5 = excellent). Regarding hepatic lesion identification, 2 investigators with 4 and 11 years of experience in abdominal MRI, respectively, recorded all visible focal hepatic lesions (≥ 5 mm, maximum 10 lesions per patient). Pathological examinations were not performed to confirm lesions identified as focal hepatic lesions. The determination of the presence of true lesions was made by 1 radiologist (study coordinator: 21 years of experience in abdominal MRI) after reviewing clinical-laboratory information, other MR sequences, and follow-up examinations.

### Quantitative analysis

Quantitative measurements were performed by one radiologist (M.T.) on a workstation (Shade Quest; FUJIFILM Medical Solutions, Tokyo, Japan). A circular or oval region of interest (ROI) (50–200 mm^2^ according to the target lesion or organ) was placed in the liver parenchyma, as well as a hepatic lesion (the largest lesions if multiple lesions were observed) at the same slice level where the hepatic lesion was present. Large vessels, bile ducts, and any artifacts were avoided to compare the noise (standard deviation: SD) of the liver, signal-to-noise ratio (SNR) of the liver, and contrast-to-noise ratio (CNR) between standard HBP imaging using CS and AiCE and high-precision HBP imaging using the modified Fast 3D mode wheel and PIQE. The SNR of the liver and CNR were calculated using the following formulas:

$${\text{SNR}} = {{{\text{signal intensity }}\left( {{\text{SI}}} \right){\text{ of liver}}} \mathord{\left/ {\vphantom {{{\text{signal intensity }}\left( {{\text{SI}}} \right){\text{ of liver}}} {\text{SD of the liver}}}} \right. \kern-0pt} {\text{SD of the liver}}}.$$

$${\text{CNR}} = {{\left( {{\text{SI of the liver}}{-}{\text{SI ofthe lesion}}} \right)} \mathord{\left/ {\vphantom {{\left( {{\text{SI of the liver}}{-}{\text{SI ofthe lesion}}} \right)} {\text{SD of the liver}}}} \right. \kern-0pt} {\text{SD of the liver}}}.$$

### Statistical analyses

Quantitative and qualitative factors were compared using the Wilcoxon signed-rank test. *p* values of < 0.05 were considered to indicate statistical significance. Inter-reader reliability was calculated using the Fleiss kappa test. The Fleiss kappa value was interpreted as follows: 0.81–1.00, excellent agreement; 0.61–0.80, substantial agreement; 0.41–0.60, moderate agreement; 0.21–0.40, fair agreement; < 0.20, poor agreement. All data were analyzed using SPSS (version 27.0; IBM, Armonk, NY, USA).

## Results

**Fig. 2:**
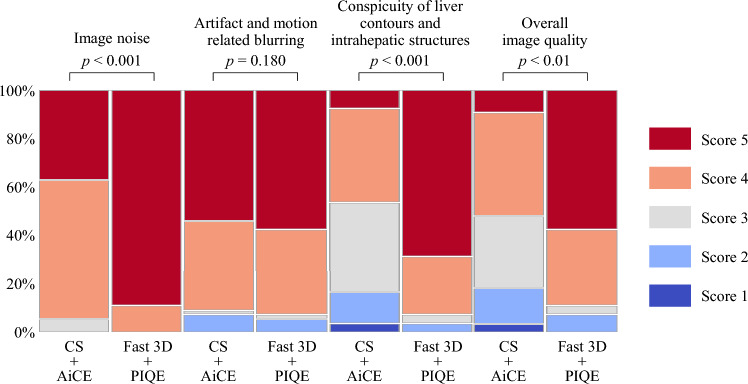
Bar charts show summary of qualitative five-grade scoring

**Fig. 3:**
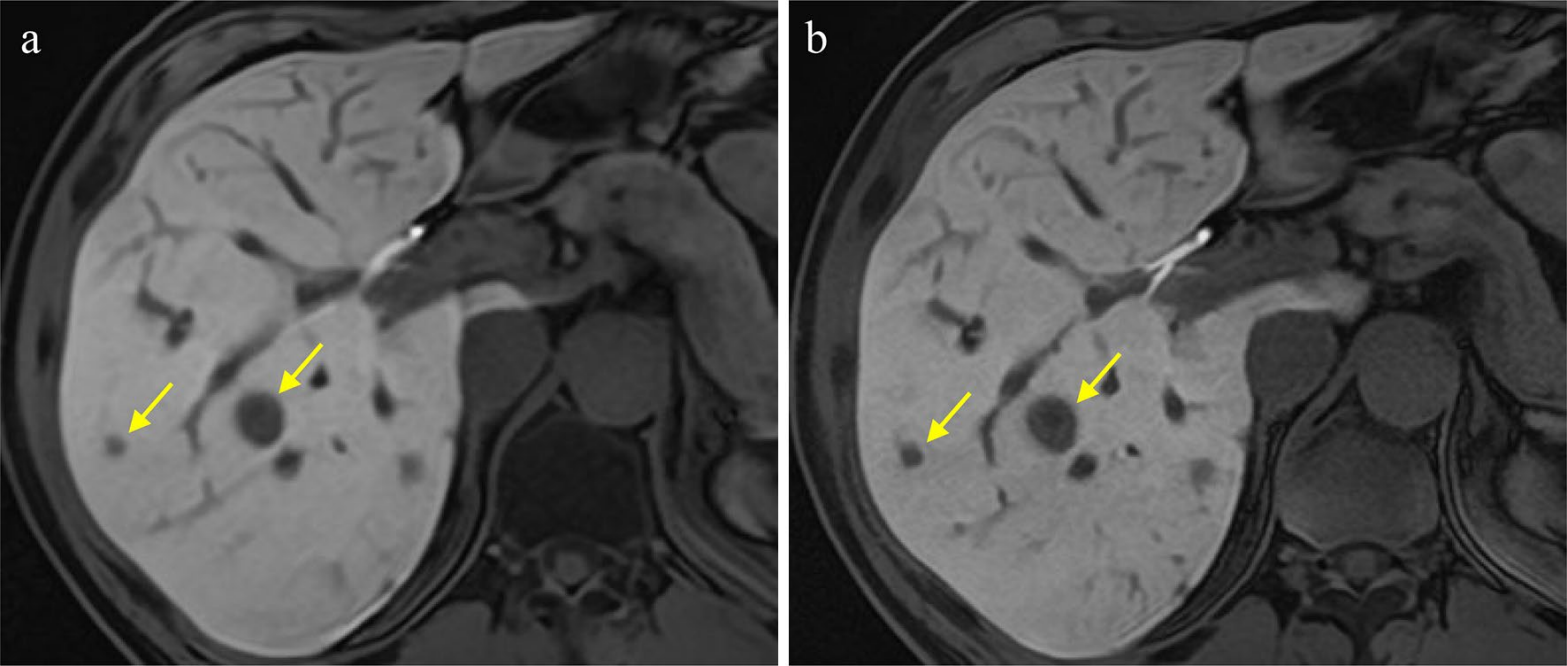
MR images in a 54-year-old man with multiple liver metastases from pancreatic cancer. **a** HBP image with CS using AiCE. **b** High-precision HBP image with modified Fast 3D mode wheel and PIQE. Conspicuity of intrahepatic vascular structures, bile duct and metastatic tumors (arrows) was better in high-precision HBP image with modified Fast 3D mode wheel and PIQE (**b**), compared with HBP image with CS using AiCE (**a**)

**Fig. 4:**
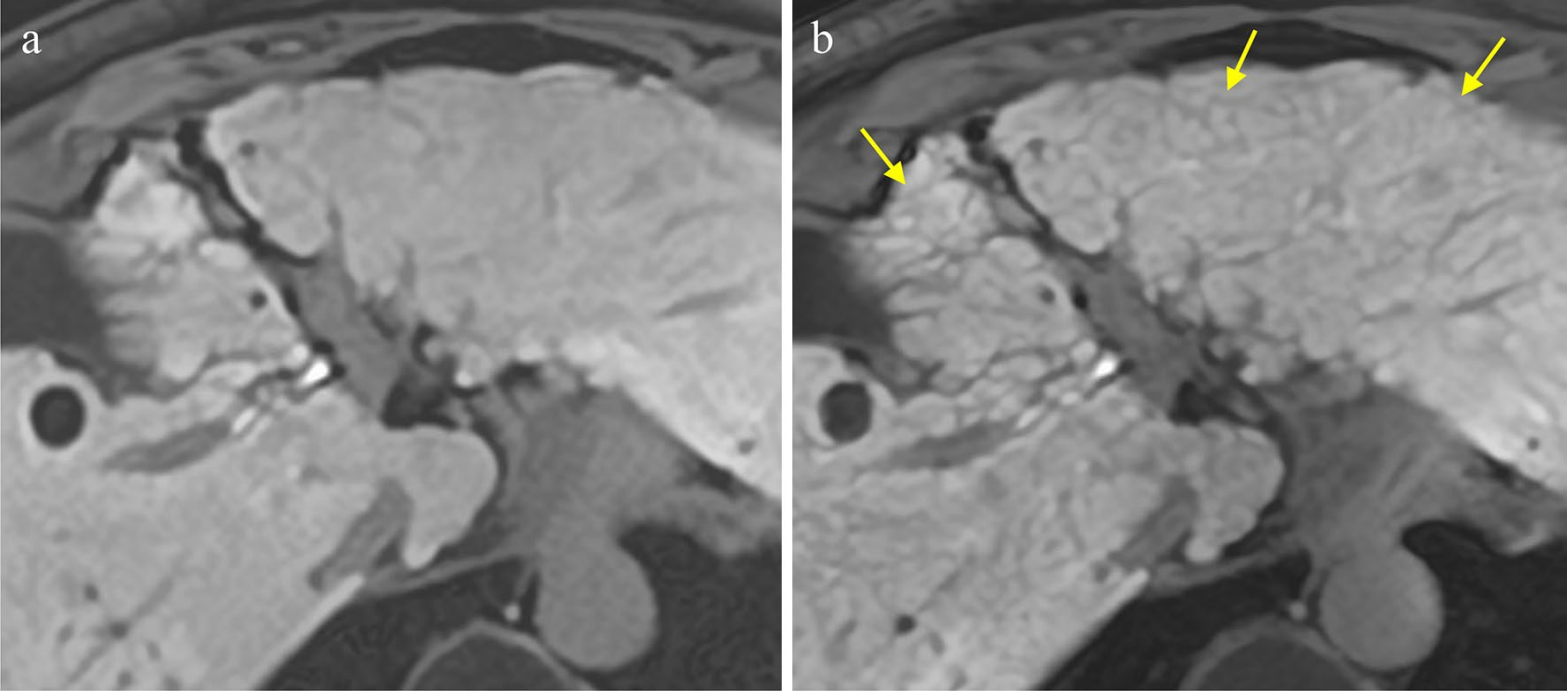
MR images in a 74-year-old woman with liver cirrhosis. **a** HBP image with CS using AiCE. **b** High-precision HBP image with modified Fast 3D mode wheel and PIQE. Hepatic regenerative nodules with hypointense thin septa (arrows) were more clearly visualized in high-precision HBP image with modified Fast 3D mode wheel and PIQE (**b**), compared with HBP image with CS using AiCE (**a**)

The results of the qualitative analysis are shown in Fig. 2. The scores for image noise, conspicuity of liver contours and intrahepatic structures, and overall image quality in high-precision HBP imaging using the modified Fast3D mode wheel and PIQE were significantly higher in comparison to standard HBP imaging using CS and AiCE (median [IQR]: 5 [0] vs. 4 [1], *p* < 0.001, 5 [1] vs. 3 [1], *p* < 0.001, 5 [1] vs. 4 [1], *p* < 0.001, respectively), while there was no significant difference in the presence of artifact- and motion-related blurring (median [IQR]: 4 [1] vs. 4 [1], *p* = 0.180) (Figs. 3, 4).

Regarding the inter-reader reliability in the qualitative analysis, the kappa values between the reviewers were moderate for image noise (0.591 [95% confidence interval {CI} 0.501, 0.682]) and artifact and motion-related blurring (0.566 [95% CI 0.484, 0.648]), substantial for conspicuity of liver contours and intrahepatic structures (0.758 [95% CI 0.692, 0.824]), and excellent for the overall image quality (0.817 [95% CI 0.751, 0.883]).

In the quantitative analysis, the noise (SD) of the liver in high-precision HBP imaging using the modified Fast 3D mode wheel and PIQE (12.6 ± 5.2) was significantly lower than that in standard HBP imaging using CS and AiCE (16.0 ± 5.4) (p < 0.001). Conversely, no significant differences were observed between high-precision HBP imaging using the modified Fast 3D mode wheel and PIQE and standard HBP imaging using CS and AiCE in terms of the SNR (mean: 31.1 ± 12.0 vs. 32.9 ± 11.0, *p* = 0.341) or CNR (mean: 15.7 ± 8.6 vs. 17.4 ± 8.1, *p* = 0.077).

For lesion detection, 259 focal hepatic lesions were evaluated in 54 patients. The mean size of these lesions was 14.0 ± 16.8 mm (range: 5–175 mm). Standard HBP images obtained using CS and AiCE detected 221 lesions with 38 false-negative cases (reader 1) and 185 lesions with 74 false-negative cases (reader 2). Conversely, high-precision HBP imaging using the modified Fast 3D mode wheel and PIQE depicted 248 lesions with 11 false-negative cases (reader 1) and 213 lesions with 46 false-negative cases (reader 2). The detection rate was 71–85% on standard HBP imaging using CS and AiCE, and 82–96% on high-precision HBP imaging using modified Fast 3D mode wheel and PIQE (Table 3).

**Table 3:** Detection of focal hepatic lesions for standard HBP images using CS and AiCE and high-precision HBP imaging using modified Fast 3D mode wheel and PIQE

	CS and AiCE		modified Fast 3D and PIQE	
	TP	FN	TP	FN
Overall				
Reader 1	221 (85)	38 (13)	248 (96)	11 (4)
Reader 2	185 (71)	74 (29)	213 (82)	46 (18)
≥ 10 mm				
Reader 1	107 (95)	6 (5)	113 (100)	0 (0)
Reader 2	100 (88)	13 (12)	108 (96)	5 (4)
< 10 mm				
Reader 1	114 (78)	32 (22)	135 (92)	11 (8)
Reader 2	85 (58)	61 (42)	105 (72)	41 (28)

*HBP* hepatobililary phase, *CS* compressed sensing, *AiCE* Advanced Intelligent Clear-IQ Engine, *PIQE* Precise IQ Engine, *TP* true-positive, *FN* false-negative

## Discussion

Our findings showed that high-precision HBP imaging using the modified Fast 3D mode wheel and PIQE had less noise and visualized the anatomical structures of the liver, including the intrahepatic vessels, more clearly in comparison to standard HBP imaging using CS and AiCE, and that the imaging technique is feasible for depicting small hepatic lesions on the HBP after contrast enhancement with Gd-EOB-DTPA. These results may be due to high-precision imaging using a combination of the modified Fast 3D mode wheel sequence and the PIQE reconstruction technique with neural networks for deep learning (i.e., denoising and up-sampling for zero-fill interpolation).

In HBP imaging with CS, previous studies have reported more subjective image noise and blurring and degradation of the overall image quality due to errors in the reconstruction steps and under-sampling of the k space inherent to the CS technique [1, 4, 10, 11]. Regarding Fast 3D mode, a “standard” wheel sequence is the technique in which both slice encoded and phase encoded data are filled from the center of the k-space, which is divided into a fan shape, and the data are finally collected in a wheel shape, and the four corners of the k-space are thinned out to reduce imaging time [12–14]. However, when the Fast 3D factor is increased, the entire wheel becomes smaller and high-frequency components are reduced, resulting in blurred images. Conversely, in the newly developed, “modified” Fast 3D mode wheel sequence, data are sequentially filled for both slice encoding and phase encoding during wheel-shaped data sampling, and data are thinned out using the asymmetric half Fourier method in the slice encoding direction, thereby reducing imaging time. The advantage of this method is that images are not blurred by changing the data-filling direction from centric to sequential. In other words, during data filling, the signal becomes unstable in the initial phase of the steady state. On the other hand, by changing to "sequential” filling, data are filled in the order of high, low and high frequency, so that the stable signal is placed in the center of the k-space, which reduces blurring and improves image quality.

Additionally, in this study, the modified Fast 3D mode wheel sequence was combined with the PIQE reconstruction technique. PIQE is a technique for reconstructing low-resolution, low-SNR images into high-resolution, high-SNR imaging using a deep-learning algorithm (9). The process flow consists of a combination of two deep learning processes (denoising and upsampling for zero-fill interpolation). First, a complex image is taken as the input and denoised by the first deep learning process. Next, the denoised image is subjected to zero filling to achieve a high resolution. Finally, the next deep learning process (upsampling) is performed to solve the blurring and ringing issues of zero filling, and the images are reconstructed as if the high-precision images are actually fully sampled. Thus, a combination of the modified Fast 3D mode wheel sequence and PIQE reconstruction contributes to resolving the trade-off between obtaining high-precision images with less image noise and reducing the acquisition time. The drawback of PIQE is its long reconstruction time. The reconstruction time of PIQE is more than twice as long as that of AiCE because of denoising and up-sampling. However, with newer MR versions, PIQE reconstruction times have been reduced.

In this study, the ability to identify focal hepatic lesions was improved by high-precision HBP imaging using the modified Fast 3D mode wheel and PIQE in comparison to standard HBP imaging using CS and AiCE. Since both intrahepatic vessels and focal hepatic lesions show low signal intensity on HBP images, it may sometimes be difficult to differentiate small focal hepatic lesions from cross-sectioned blood vessels. Since continuity with blood vessels is an important differentiating point, high-precision HBP imaging using the modified Fast 3D mode wheel and PIQE, with its superior conspicuity of the intrahepatic structures, is thought to improve lesion identification and reduce false negatives. Furthermore, small focal hepatic lesions tended to be invisible on standard HBP imaging using CS and AiCE because the contours of the lesion were blurred and the conspicuity was diminished, resulting in an increase in false negatives.

The present study was associated with several limitations. First, a selection bias may have existed due to the retrospective design and relatively small sample size. Second, the patient population in this study was heterogeneous and included patients with various types of focal hepatic lesions. However, this may be of practical value in evaluating the utility of high-precision HBP imaging using the modified fast 3D mode wheel and PIQE in routine clinical practice, where a variety of lesions may be encountered. Third, the MR system used in this study did not support the combined use of CS and PIQE in the BH 3DT1-weighted GRE sequence. Therefore, new high-precision HBP images could not be compared to HBP images obtained using CS and PIQE. Finally, pathological examinations were not performed to confirm the focal hepatic lesions identified in this study. Therefore, we did not evaluate the diagnostic ability for differentiating focal hepatic lesions. However, we focused on the diagnostic performance in lesion identification rather than lesion characterization. Further studies with larger populations are required to validate our results.

In conclusion, the study demonstrated the feasibility of applying high-precision MR sequences obtained using the modified Fast 3D mode wheel and PIQE for the HBP of BH 3D T1-weighted GRE imaging.

## Abbreviations

AiCE: Advanced Intelligent Clear-IQ Engine
BH: Breath-hold
CNR: Contrast-to-noise ratio
CS: Compressed sensing
HBP: Hepatobiliary phase
PIQE: Precise IQ Engine
SNR: Signal-to-noise ratio
